# Temporal evolution of a seismic sequence induced by a gas injection in the Eastern coast of Spain

**DOI:** 10.1038/s41598-017-02773-2

**Published:** 2017-06-06

**Authors:** S. Ruiz-Barajas, N. Sharma, V. Convertito, A. Zollo, B. Benito

**Affiliations:** 1 Dpto. Ingeniería Topográfica y Cartografía, Universidad Politécnica de Madrid, Madrid, Spain; 2 Dpto. Fisica, Università degli Studi Federico II, Napoli, Italy; 3 Istituto Nazionale di Geofisica e Vulcanologia Osservatorio Vesuviano, Napoli, Italy; 4 Shillong Geophysical Research Centre, Indian Institute of Geomagnetism, Shillong, India

## Abstract

Induced seismicity associated with energy production is becoming an increasingly important issue worldwide for the hazard it poses to the exposed population and structures. We analyze one of the rare cases of induced seismicity associated with the underwater gas storage operations observed in the Castor platform, located in the Valencia gulf, east Spain, near a complex and important geological structure. In September 2013, some gas injection operations started at Castor, producing a series of seismic events around the reservoir area. The larger magnitude events (up to 4.2) took place some days after the end of the injection, with EMS intensities in coastal towns up to degree III. In this work, the seismic sequence is analyzed with the aim of detecting changes in statistical parameters describing the earthquake occurrence before and after the injection and identifying possible proxies to be used for monitoring the sequence evolution. Moreover, we explore the potential predictability of these statistical parameters which can be used to control the field operations in injection/storage fluid reservoirs. We firstly perform a retrospective approach and next a perspective analysis. We use different techniques for estimating the value of the expected maximum magnitude that can occur due to antropogenic activities in Castor.

## Introduction

Induced seismicity typically refers to low magnitude earthquakes (usually M_w_ < 5.0), that are caused by human activities which alter the stress field of the Earth’s crust^1^. According to Ellsworth^2^ the induced seismicity may include both the earthquakes that primarily release stresses created by the fluid injection (or other industrial activity) in the near volume surrounding the downhole injection point and triggered events that release tectonic stresses as, for instance, an effect of pore pressure increase at critically stressed faults. This kind of seismicity has been observed in relation to changes in the dam water level, hydro-fracturing operations, fluid injections and extraction in geothermal areas^2–4^. Historically, some best-documented and well-known cases of induced seismicity related to injection of fluids into the crust have been provided (e.g., the Denver earthquakes as reported by Healy *et al*.^5^; Simpson^6^). Recently, energy development technologies that involve fluid injection operations are being increasingly used in different projects worldwide and different cases of injection-induced seismicity have been reported. However, it is rare to find cases of induced seismicity associated with underwater gas storage operations.

**Figure 1:**
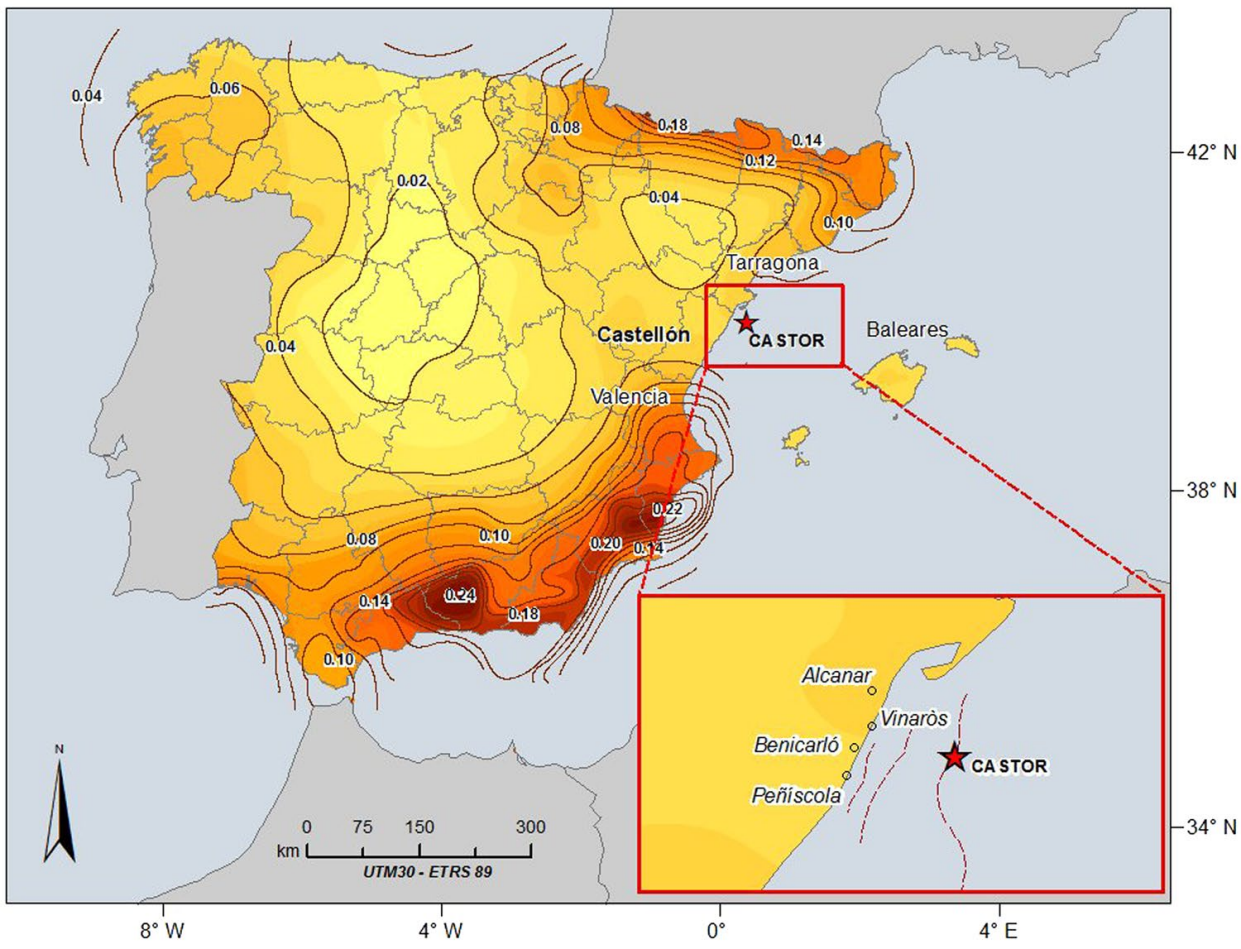
Castor platform location and the hazard map for return period of 475 years. The latter has been obtained in a recent study developed by the Polytechnic University of Madrid and the Spanish IGN aimed at the revision of the Spanish building code. The map shows the horizontal values of PGA on rock sites having 10% of probability of exceedance in 50 years (IGN-UPM, 2013^8^). The figure was generated by using the ESRI software, ArcGIS for desktop, version 10.1.

In the present study we analyze one of the rare and best recorded examples of seismic sequences induced by gas storage. In particular, we focus on Castor field, which is the biggest underground reservoir of natural gas in Spain, located in the Gulf of Valencia (NE Mediterranean) (Fig. 1). The offshore platform is located 22 km away from the nearest inland town (Vinaroz, Castellón). The target reservoir is contained within a fault-limited, horst structure with an average depth of more than 1700 m below the mean sea level. The main fault of this structure is the Amposta fault.

The recorded seismicity of the zone, before the gas injection operations, is the lowest in the eastern Spain, where only two earthquakes with magnitudes larger or equal to M_w_ 5.0 occurred (1885, M = 5.4 and 1995, M = 5.0) at distances greater than 50 km from the reservoir.

After the beginning of injection in September 2013, a sequence of seismic events occurred, reaching a maximum magnitude of 4.2 and macroseismic intensities in coastal towns of degree III on the EMS-98 scale. While the first part of the sequence can be directly related to the injection process, Cesca *et al*.^7^ argue that the second part is of tectonic origin. The idea is that the seismic cycle of critically pre-stressed faults has been shortened by the pore-pressure perturbation, thereby anticipating the occurrence of tectonic events. Nevertheless, the authors pointed out that a natural cause for the earthquakes cannot be completely excluded, although, according to the historical seismicity in the region it is unlikely.

Other than the origin of the induced seismicity, the challenging issues to be faced in order to reduce its associated hazard, concern the possibility to predict their space-time evolution and to estimate the maximum expected magnitude during the field operations. Thus, in this study we first analyze the space-time evolution of the observed seismicity in the Castor area during and after the injection. The aim is to examine the statistical parameters of the two clusters and to identify possible proxies, which can be used to detect significant seismicity changes occurring during the injection operations. Next, we explore the potential predictability of some statistical parameters that could be used to guide the field operations in near real-time in order to manage future emergencies. We tackle this objective carrying out a time-dependent seismicity parameters estimation. Furthermore, we estimate the expected maximum magnitude by applying the procedures proposed by Kijko^9^.

It is important to note that the data used in the present study is limited because of the constrained accessibility to the whole database acquired during the operation by local networks. Thus, only the public data provided by the Spanish National Network belonging to IGN (National Geographic Institute of Spain) are therefore used for the analysis. We emphasize that even with this limited data set, relevant results can be obtained in order to characterize the statistical behavior of the induced seismicity at this site or where local data are also available.

## Castor Project

Castor gas storage is one of the most important investments made by the Spanish gas system to guarantee supply and price stability for the future. The Castor project seeks to build an underground natural storage for gas in the already exploited Amposta oil reservoir, an offshore reservoir located in the Valencia Trough, being the settlement of Vinaroz the nearest inland town.

The project consists of a fixed offshore platform connected with an inland platform by a gas pipeline which is 30 km long. The plant facilities take the gas from the general gas network for storage in the submarine underground. To do so, the gas is transferred via pipeline, mostly running under the sea, which connects the Ignacio Pérez land plant (located in the town of Vinaroz) to a platform which is located 22 km offshore. When the gas system requires it, these facilities would be able to return gas from the storage in the same conditions as it was received through the same submarine gas pipeline.

The total gas storage capacity of the reservoir is 1900 millions of cubic meters (1300 Mm^3^ of working gas and the 600 Mm^3^ of cushion gas). The injection capacity is 8 Mm^3^/day and the deliverability rate (withdrawal rate) is 25 Mm^3^/day. This quantity could meet the gas consumption in Spain for 50 days^10^.

On September 5^th^2013, three days after the beginning of the gas injection, a seismic sequence with an important number of micro-earthquakes started. Some of the events were felt by the population, creating a great public concern. Therefore, on September 16^th^2013 the gas injection was stopped. Nevertheless, some events were still being recorded, reaching the larger magnitudes, M_w_ 4.1 and 4.2, during the first week of October. According to the IGN data, a total of sixteen events had an assigned EMS intensity value, reaching a grade of III in some coastal settlements (Table 1). A year later (on October 4^th^2014), after different risk assessments and according with geological and seismological reports, the Spanish government approved a royal decree for the temporary halting of the facility (hibernation period). Moreover, it was warned that this situation could be reverted for “close, dismantle or exploit the facility without danger of perceptible earthquake” in the future if this was advised upon specific technical studies^11^.

**Table 1:** Events occurred after the injection with an assigned intensity value (IGN).

ID	DATE	HOUR	LAT	LONG	Depth (km)	I (EMS)	M	^M^_TYPE_
1234647	24/09/2013	0:21:50	40.3805	0.7142	3	II	3.6	mb_Lg_
1236155	29/09/2013	16:36:23	40.3836	0.6977	3	II	3.4	mb_Lg_
1236342	29/09/2013	21:15:05	40.4016	0.7085	2	II	3.2	mb_Lg_
1236361	29/09/2013	21:23:16	40.3858	0.6995	6	II	3.1	mb_Lg_
1236429	29/09/2013	22:15:48	40.3849	0.708	3	III	3.7	mb_Lg_
1236581	30/09/2013	2:21:16	40.3821	0.715	1	III	3.9	Mw
1237104	01/10/2013	3:32:44	40.3914	0.7129	1	III	4.2	Mw
1237788	02/10/2013	9:27:26	40.4025	0.7048	4	II	3.0	mb_Lg_
1237944	02/10/2013	23:06:49	40.399	0.7044	2	III	4.1	Mw
1237976	02/10/2013	23:29:29	40.4031	0.7205	3	III	4.1	Mw
1238106	03/10/2013	3:13:02	40.4046	0.7069	3	II	3.2	mb_Lg_
1238333	04/10/2013	8:49:48	40.3932	0.7121	4	III	3.7	mb_Lg_
1238401	04/10/2013	9:55:19	40.3876	0.7164	4	II	3.5	Mw
1238531	04/10/2013	12:56:10	40.401	0.6903	1	II	3.1	mb_Lg_
1238635	04/10/2013	20:02:24	40.3862	0.6989	2	II	3.3	mb_Lg_
1241021	15/10/2013	21:16:18	40.3745	0.7325	4	I/II	1.8	mb_Lg_

## Geological and tectonic environment of Castor

Castor is located inside a regional rift structure (Valencia Basin) caused by stretching of the lithosphere in the complex context linked with the collision between the African and Eurasian continental plates. The Valencia trough extends to the NE by the Gulf of Lions, reaching the whole length of nearly 600 km and a width of 200 km up to the Baleares Island.

**Figure 2:**
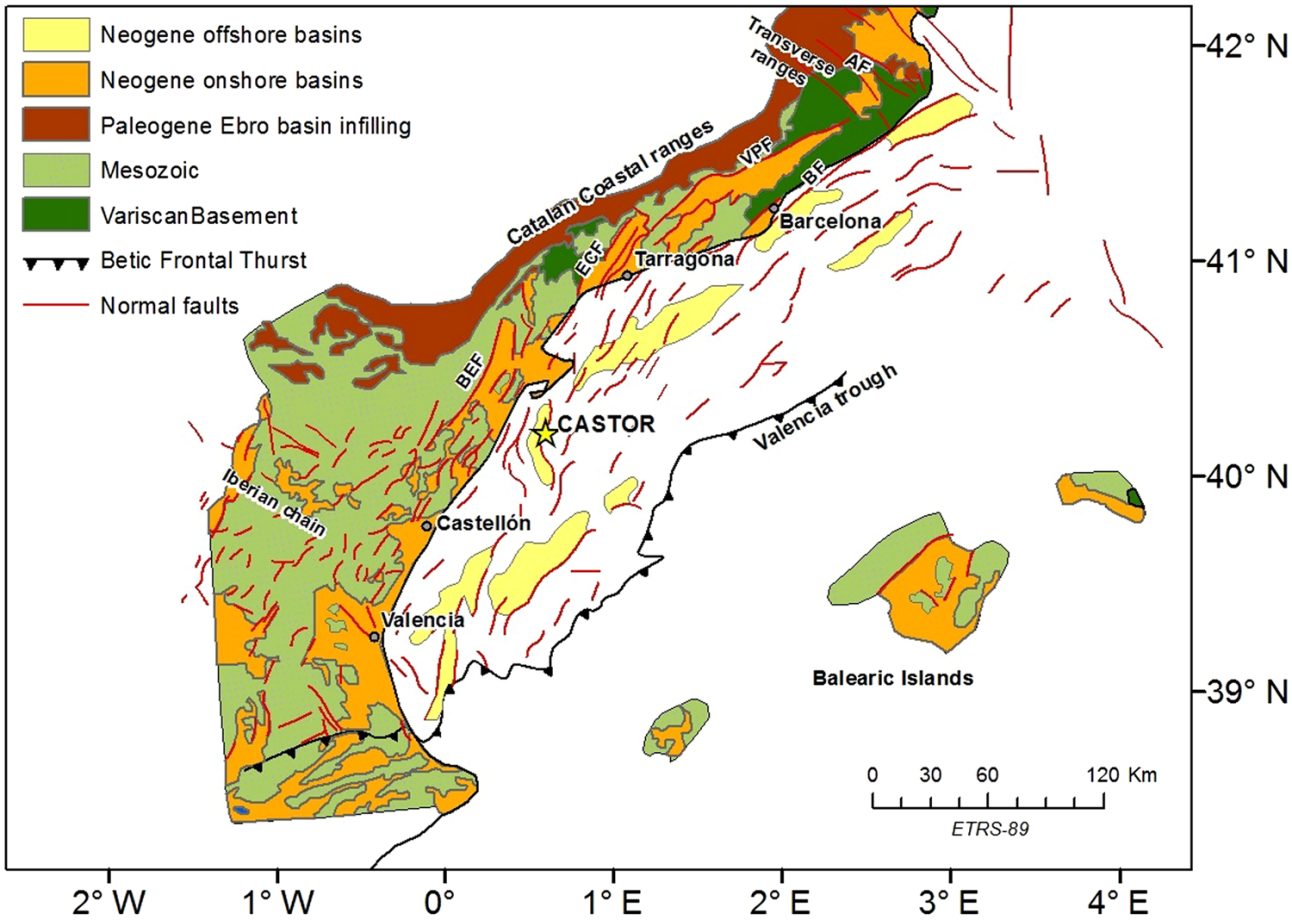
Simplified geological framework around the Castor platform. AF: Amer fault; BF: Barcelona fault; VPF: Vallès-Penedès fault; ECF: El Camp fault; BEF: Baix Ebre fault. The figure was generated by using the ESRI software, ArcGIS for desktop, version 10.1. Data source: Perea^15^. Based on Roca^16^ and Roca and Guimerà^17^.

The trough displays a well-developed horst and graben structure with a general NE-SW trend, where Pre-Neogene tectonic structures are difficult to be recognized^12^. In this tectonic setting, the deformation is partitioned into two families of faults: one is oriented in NNE-SSW and other is in NNW-SSE direction which also coincides with traces of greater length, mapped at a QAFI (Quaternary Active Faults of Iberia) database^13^). Castor is located in an area where these fault segments interact, localized the longest failures between the platform and the coast (Fig. 2). From published information regarding the potential seismogenic faults, we can establish that no all these structures can be considered as active faults; however some of them affect the upper Miocene and Quaternary formations. Therefore they have the potential to reactivate seismicity due to changes of induced stresses.

The longest fault of the system, that is the Amposta Fault, is considered as the main structure to be taking into account in the estimation of seismic hazard. The length and continuity of the fault trace, as well as its geometry at depth are the key aspects to assess its seismogenic capacity because they determine the fault size and consequently the energy that can be released during an earthquake slip event. In the QAFI database the fault appears as a segment of 51 kilometers in length, with a pane that reaches a depth of 15 kilometers. In the central part, the fault turns towards NNE-SSW direction and from that section, several NNW-SSE branches appear.

However the tectonic model of the area is complicated by the presence of other secondary faults, shorter and jump, but they can play an important role in the interpretation of the seismicity. Numerous studies have been published about the activity, geometry and kinematic parameters of these faults^14–17^. From the extracted information it can be concluded that 12 active faults are documented on land in a radius of 120 km around the site of Castor, containing 23 segments whose slip rates ranges from 0.01 mm/y to 0.15 mm/y, with recurrence intervals between 2.900 and 50.000 years.

The raising question is which fault gets triggered after the injection and its seismogenic potential to produce earthquakes with magnitudes larger than the observed ones. The location of the events suggests that a secondary fault was activated during the 2013 earthquake sequence with a rupture length of 3 km, instead of the Amposta fault which a priori seemed the main candidate^7^. Nevertheless, in order to answer this question more high quality data are required, which are not currently available.

## Seismicity and seismic hazard around the Castor platform

**Figure 3:**
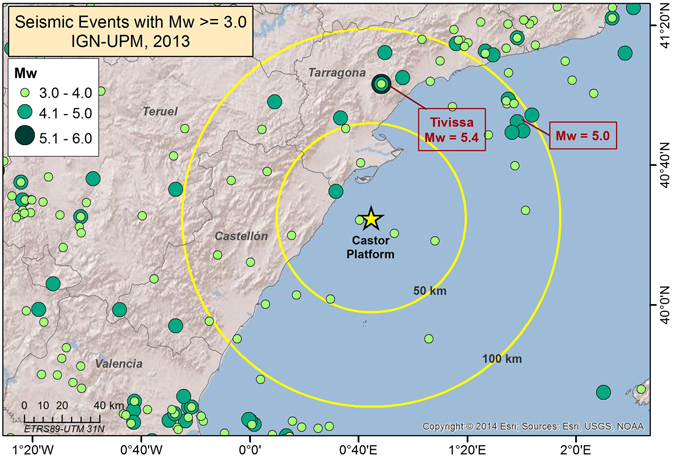
Historical and instrumental seismicity in the area of 100 km around Castor. Time period: 1048-2013 (Data source: IGN^19^). The figure was generated by using the ESRI software, ArcGIS for desktop, version 10.1.

The area where the Castor platform is located is one of the lowest seismic hazard sites in the Community of Valencia (East Spain). Before the injection operations, very few low magnitude earthquakes had been recorded in the area within a radius of 50 km around the reservoir (Fig. 3 and Table 2). Based on the IGN catalogue, only 11 earthquakes with M > 4.0 have occurred during the last 110 years before the injection in a radius of 100 km with respect to location of Castor platform, thus indicating an approximate rate of 0.1 events per year with magnitude M > 4.0. On the other hand, the 2013 Castor sequence showed a seismicity rate much higher than this background level (e.g. four M about 4.0 events in less than one month) which clearly indicates a strong correlation between injection operations and seismicity. Figure 3 shows the historical and instrumental seismicity in the area SE Spain, highlighting the seismicity included between the radius of 50 and 100 km around the platform.

**Table 2:** Location and size parameters corresponding to earthquakes with epicenters in the area of 100 km (M_w_ ≥ 4.0) around Castor platform.

Date			Localization		M_w_		Original size Parameter	
Day	Month	Year	Long	Lat	M_w_	^σ^_Mw_	Type and value	Source
17	11	1879	0.4833	40.5167	4.3	0.8	I (EMS) V	IGN
21	11	1755	0.0833	40.9333	4.1	0.8	I (EMS) IV–V	IGN
3	10	1845	0.7500	41.0333	5.4	0.5	I (EMS) VII	ICC
7	10	1845	0.7500	41.0333	4.9	0.5	I (EMS) VI	ICC
26	1	1917	0.7667	41.1833	4.3	0.3	I (EMS) V	ICC
2	6	1930	1.000	41.2500	4.0	0.4	mD-MMS 3.9	IGN
11	11	1932	0.5000	40.8667	4.3	0.5	I (EMS) V	IGN
13	2	1949	0.8833	41.0667	4.1	0.4	mD 4.0	IGN
24	8	1987	1.5517	40.9733	4.1	0.3	mb﻿_Lg_ 4.0	IGN
15	5	1995	1.6100	40.8667	5.0	0.3	mb_Lg_ 4.9	IGN
15	5	1995	1.5783	40.8167	4.4	0.3	mb﻿_Lg_ 4.3	IGN

The location and size of the earthquakes recorded or reported in a radius of 100 km with respect to Castor platform, with M_w_ 4.0 are summarized in Table 2. Data are extracted from the catalog elaborated for a recent seismic hazard assessment study for Spain, aimed at the revision of the Spanish Earthquake-Resistant Building Code^8, 18^. It is important to note that only two earthquakes are reported in this area with a magnitude larger or equal to 5.0: the Tivissa earthquake, occurred on October 3^rd^ 1885, with an MSK intensity I = VII (and estimated magnitude M_w_ = 5.4) and another earthquake which took place on May 5^th^1995 in the Catalonian Coast with magnitude M_w_ = 5.0.

Due to the low seismicity level, the resulting seismic hazard in the area where the Castor gas storage is located (Castellón province), is the lowest of Eastern Spain. Nevertheless, the hazard of the surrounding areas (Murcia, Valencia and Alicante) is one of the highest in the country^5^, (see Fig. 1).

## Seismic Data

**Figure 4:**
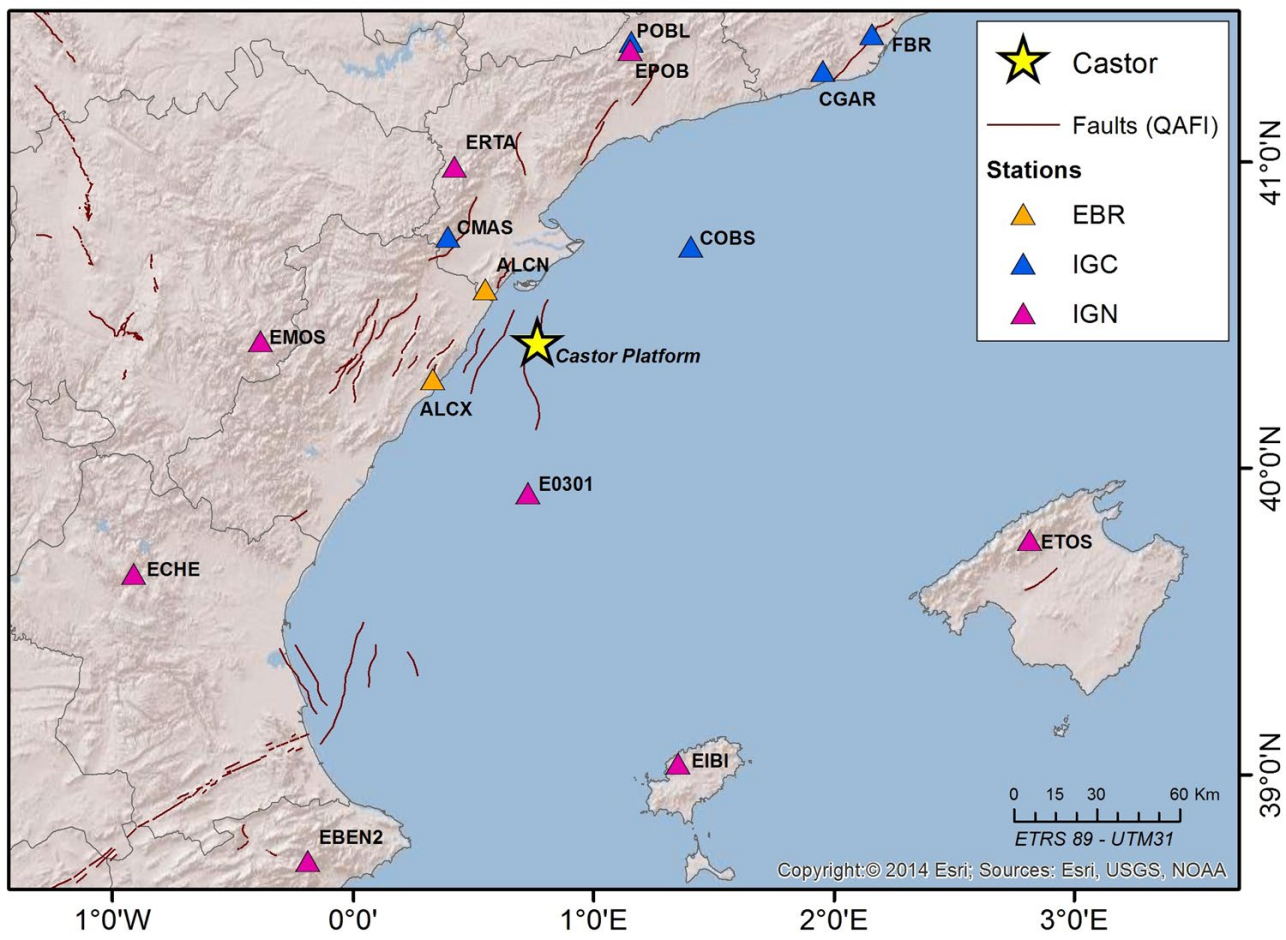
Seismic stations close to Castor platform, which recorded events of the seismic sequence after the injection. The stations are managed by different institutions: Ebro Observatory stations (EBR) in orange, Catalonian Geological Institute (IGC) stations in blue and Spanish National Network (IGN) in pink. The figure was generated by using the ESRI software, ArcGIS for desktop, version 10.1.

Data used in this study are those recorded by stations managed by the IGN, which at the time of the present study are the only publicly available data. This fact involves a limitation in our database, because the seismic network of the IGN has a strong asymmetry and azimuthal gap with a lack of close stations, being the nearest one located 55 km far from the Castor platform (Fig. 4).

In the recent study of Cesca *et al.* ^7^, the whole catalogue from the Ebro Observatory was used, which contains 1002 earthquakes, 982 of them with local magnitude assigned (between 0.0 and 4.0). They provided an absolute and relative location of these events applying full-waveform inversion techniques.

The IGN catalog used in this study contains 552 events recorded from 5^th^ September to 29^th^ October 2013. Most of the events have been provided with magnitude mb_Lg_, while for larger magnitude events (M 3.5) the moment magnitude M_w_ has been directly assigned. In order to avoid mixing different magnitude scales, we have homogenized the catalog to moment magnitude M_w_ applying the relationship used in the recent Seismic Hazard map of Spain^8, 18^:

1 $${M}_{w}=0.676+0.836\,m{b}_{Lg}$$

**Figure 5:**
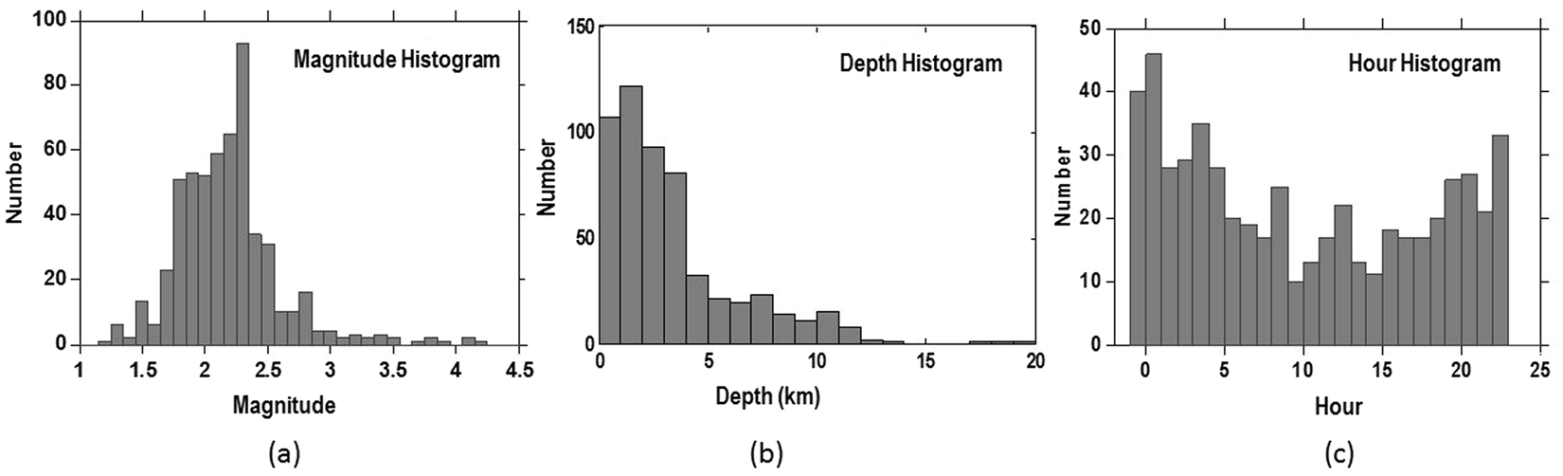
Histograms of the whole seismic sequence analyzed in this study; (**a**) magnitude distribution of events; (**b**) depth distribution of the events; (**c**) hour distribution of events. The figure was generated by using the ZMAP software.

The moment magnitude of the homogenized catalogue ranges between M_w_ 1.2 and M_w_ 4.2. Most of the events are located in the upper 10 km and were detected during night-time hours (Fig. 5), probably due to the less noise during night.

## Time and spatial evolution of the seismicity

The operations of gas injection in the Castor platform started on 2^nd^ September 2013. Three days later, a few earthquakes of magnitude around M_w_ 1.6 were recorded in the area.

On 9^th^ September 2013, the event rate started to increase. The phenomenon continued until the maximum rate of 13 to 24 events per day was reached by 13^th^ September. The magnitude of the events also increased with the frequency of occurrence and maximum observed magnitude was M_w_ 3.2 on 13^th^ September.

**Figure 6:**
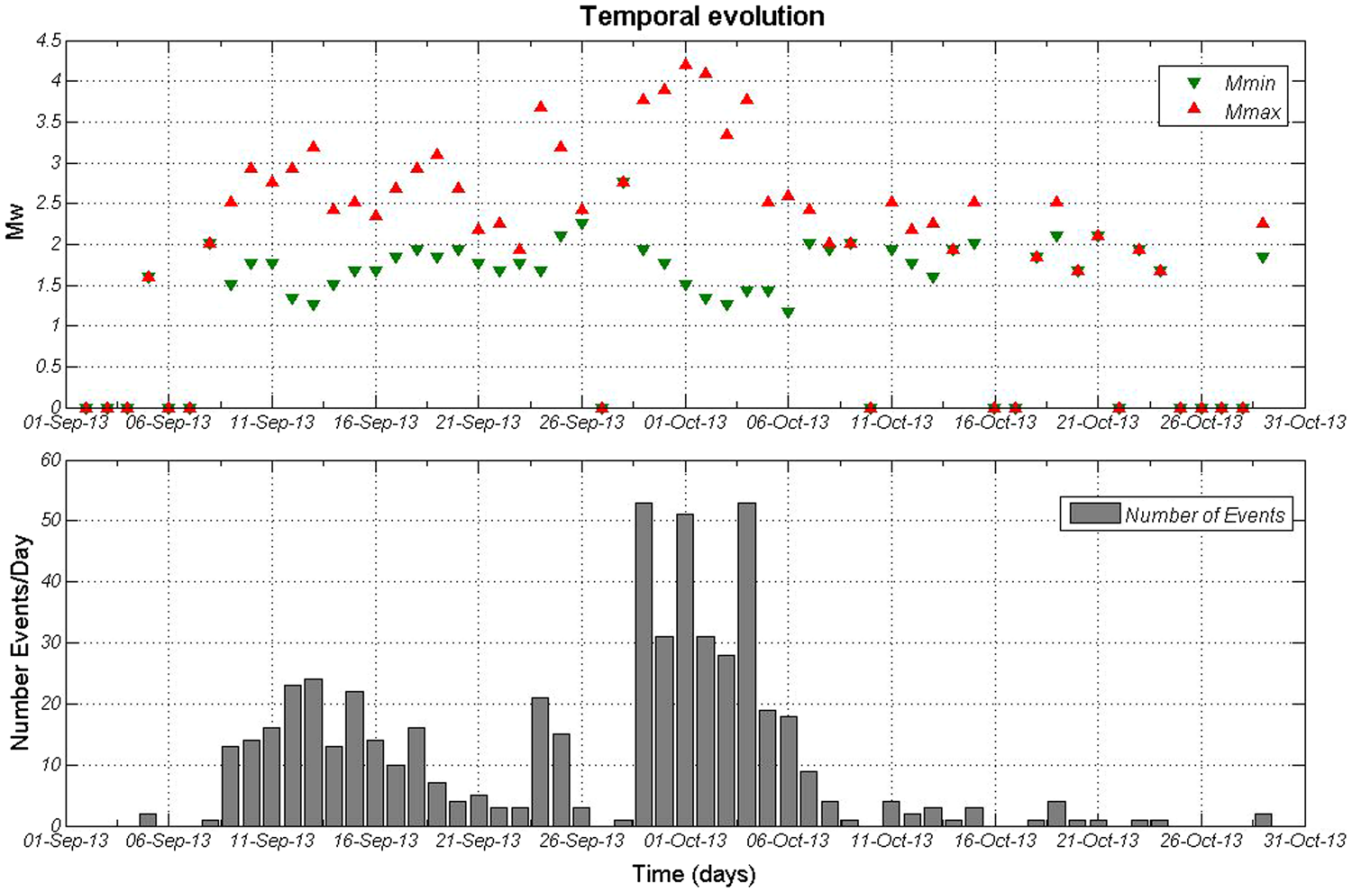
Temporal evolution of the sequence analyzed in this study. Red and green triangles correspond to the observed maximum magnitude and minimum magnitude per day, respectively and each gray bar represents the number of events per day.The figure was generated by using the ZMAP software.

Due to this increase in the seismicity level, both in terms of rate and magnitude, a warning was issued to communities living in the coastal areas. As a consequence, on 16^th^ September 2013 the injection operations were stopped. A total volume of 1.02 * 10^8^ m^3^ of gas had been injected^7^ since the operations started through a 1750 m-deep well. The number of events recorded during the injection period was 142. However, as it is shown in Fig. 6, many events were still being detected a few days after the halt of the gas injection. Indeed, during the last days of September, the number of events considerably increased and, at the beginning of October 2013, two events of magnitude larger than M_w_ 4.0 occurred (M_w_ 4.2 on 1^st^ October 2013 and M_w_ 4.1 on 2^nd^ October 2013).

**Figure 7:**
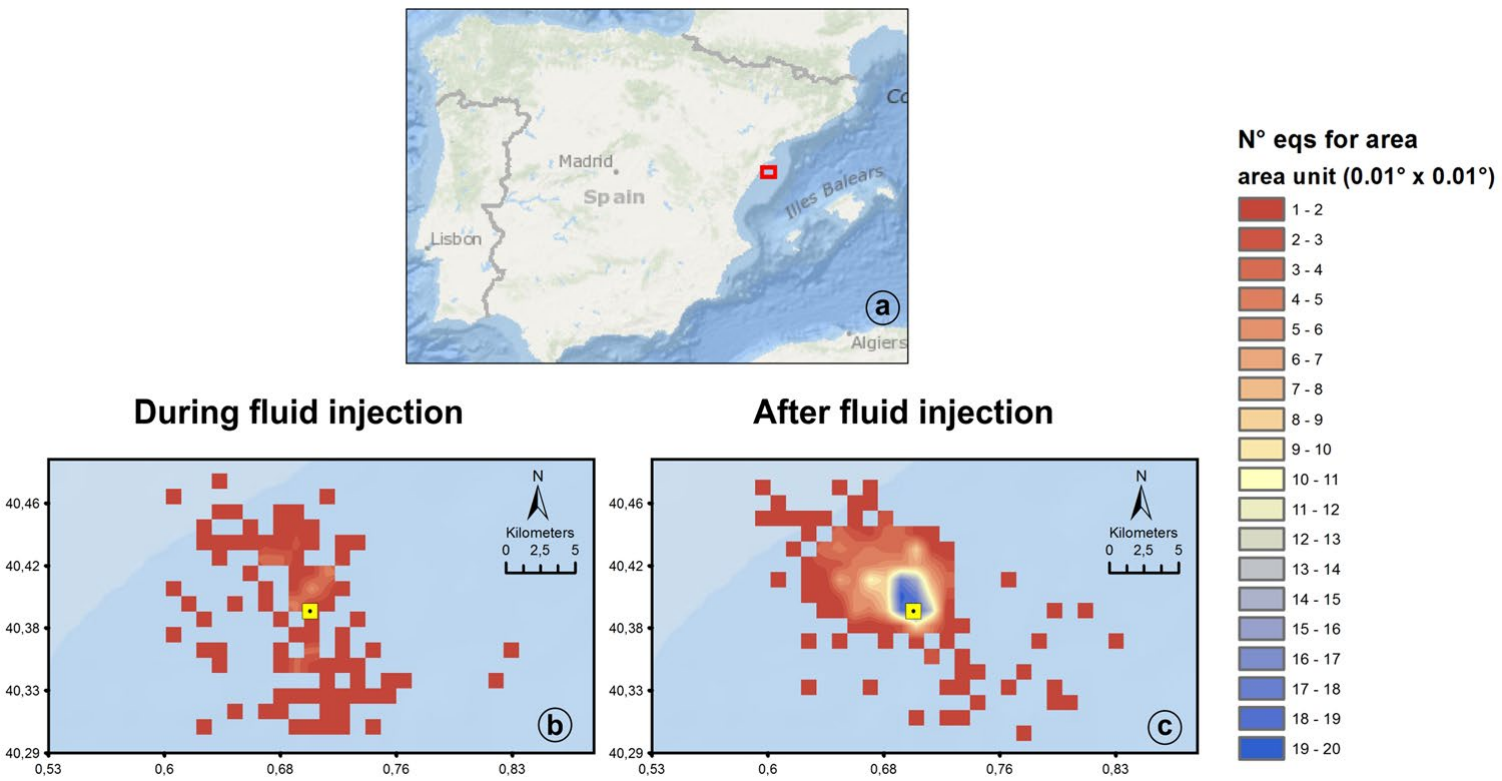
(**a**) shows the location map of the study area. In (**b**) and (**c**), the number of earthquakes for area unit during and after the fluid injection is displayed, respectively. The area unit is 0.01 × 0.01 decimal degrees. The Castor platform is indicated by yellow square. The figure was generated by using the ESRI software, ArcGIS for desktop, version 10.1.

**Figure 8:**
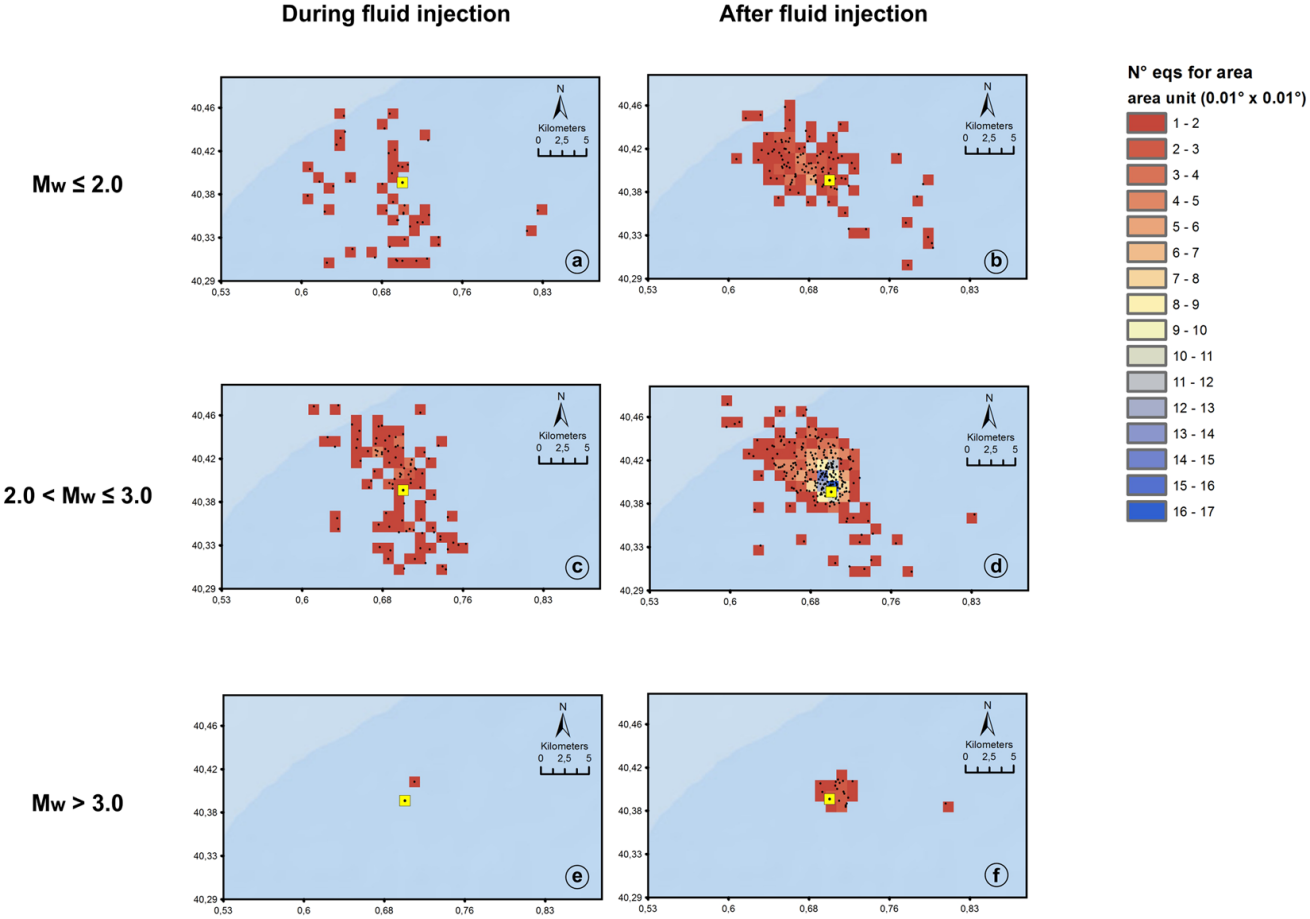
Number of earthquakes for area unit during (**a**,**c**,**e**) and after (**b**,**d**,**f**) the fluid injection. The area unit is 0.01 × 0.01 decimal degrees. The earthquakes are showed with black points and are divided in magnitude classes. (**a**) and (**b**) correspond to M_w_ 2.0 (**c**) and (**d**) correspond to M_w_ between 2.0 and 3.0. (**e**) and (**f**) correspond to M_w_ greater than 3.0. The Castor platform is indicated by yellow square. The figure was generated by using the ESRI software, ArcGIS for desktop, version 10.1.

Figure 7 shows the spatial evolution of the sequence, differentiating the events between those occurring during and after injection. We can clearly see the increase in the number of earthquakes per area after the halt of injection around the north part of the platform. In Fig. 8, the number of events per area are divided in different magnitude ranges for the two analyzed phases. As in the Fig. 7, we evidence an increase in the seismic activity around the platform after the halt of the injection for all the magnitude ranges considered. This increase in the number of earthquakes per area is more pronounced for the magnitudes between 2.0 and 3.0.

## Methods and Results

### Retrospective analysis: fitting the overall parameters of the sequence

In the first step, the seismic sequence is analyzed in a retrospective approach. In this case the analysis is carried out taking into account the whole sequence. In a second step, the seismic sequence is analyzed by considering two different, consecutive periods: during injection and after injection, so as to assess and identify possible differences that could provide possible indicators for the discrimination between induced and triggered seismicity. The software package ZMAP^20^ is used to carry out most of the analysis.

As for the Gutenberg-Richter (GR) relationship ﻿(log N = a - bm, being N = number of events with magnitude ≥ m), the a-value and the b-value parameters are calculated by using maximum-likelihood approach^21^. Moreover, three different methods are used to estimate the minimum magnitude of completeness (M_c_). These three methods are: the entire magnitude range (EMR)^22^, the maximum curvature and the goodness-of-fit^23^, all of them available in the ZMAP code^22^. A brief overview of the used methods is given in Appendix B.

The Entire Magnitude Range method (EMR) uses the whole data set and consists of two analysis phases: one to model the complete part (where it is presumed a power law behavior), and one to sample the incomplete part of the frequency-magnitude distribution (where it is assumed a normal cumulative distribution function)^22^. The maximum curvature method proposed by ref. 23 uses the maximum value of the first derivative of the frequency magnitude curve to estimate Mc. The goodness-of-fit test compares the observed frequency-magnitude distribution with synthetic ones^23^.

The results obtained by applying the three methods are summarized in Table 3. Regardless of the applied method, we observed a clear decrease in b-value when compared the b-value during injection (b ≈ 2.0 ± 0.3) with b-value obtained after the halt of injection (b ≈ 1.1 ± 0.1), whereas the b-value for the whole sequence is b ≈ 1.3 ± 0.1. The data used for obtaining the Gutenberg-Richter relationships for the three periods are reported in Figure S1 in the SI, along with the best-fitting curves and parameter estimates. A similar decrease was observed by Bachmann *et al*.^3^ during injection operations at the Basel Enhanced Geothermal System site, with a relatively high b-value (b ≈ 1.58) measured during the injection and a lower value (b ≈ 1.15) after the halt of injection.

**Table 3:** Summary of GR parameters and minimum magnitude of completeness (M_c_) obtained applying three different methods: EMR, maximum curvature and goodness-of-fit. τ (annual) represents the﻿ annual rate of events with M ≥ M_c_.

		b	*σ*_*b*_	a	τ (annual)	*M*_*c*_ ± σ
EMR	Whole sequence	1.2	0.1	5.2	6.0	2.2 ± 0.10
	During injection	1.9	0.3	6.1	7.6	2.3 ± 0.11
	After injection	1.1	0.1	4.9	5.8	2.2 ± 0.11
Maximum curvature	Whole sequence	1.3	0.1	5.3	6.2	2.3 ± 0.02
	During injection	1.9	0.2	6.2	7.7	2.3 ± 0.09
	After injection	1.1	0.1	4.8	5.7	2.2 ± 0.08
Goodness-of-fit	Whole sequence	1.2	0.1	5.1	5.9	2.3 ± 0.13
	During injection	2.0	0.3	6.3	7.8	2.3 ± 0.09
	After injection	1.1	0.1	4.8	5.8	2.2 ± 0.16

The values of M_c_ are consistent among the different used methods of estimation within ± 0.1 (a summary description of methods is given in SI).

The M_c_ value does not show important changes between the two considered periods, thus allowing us to compare the estimates of statistical parameters for the two analyzed periods. The measured M_c_ value is 2.2 ± 0.1 for the whole sequence as well as for the analysis done during and after the injection. This also confirms that M_c_ is related to the recording instruments and network geometry. The similarity of the results of both the GR parameters and M_c_ achieved with the three adopted methods, guaranties the robustness and reliability of the obtained results.

In order to characterize the temporal correlation of the events, we analyze their inter-arrival time distribution. In particular, to evaluate the clustering behavior of the seismic sequence, we use the coefficient of variation CV, which corresponds to the ratio between the standard deviation and the mean of the inter-arrival times. As shown by Kuehn *et al*.^24^ and references therein, a standard Poisson process is characterized by CV = 1; CV values greater than 1 indicate that the events tend to cluster in time with the possibility to identify a main event, whereas CV values less than 1 are characteristic of swarm type activity. The events occurred during and after the gas injection at Castor show a similar clustering behavior, being the CV in both cases greater than 1 (CV = 2.7 and CV = 3.5, respectively). So, we cannot use CV as a useful parameter to discriminate possible differences in the seismicity recorded during and after the stop of the injection.

**Figure 9:**
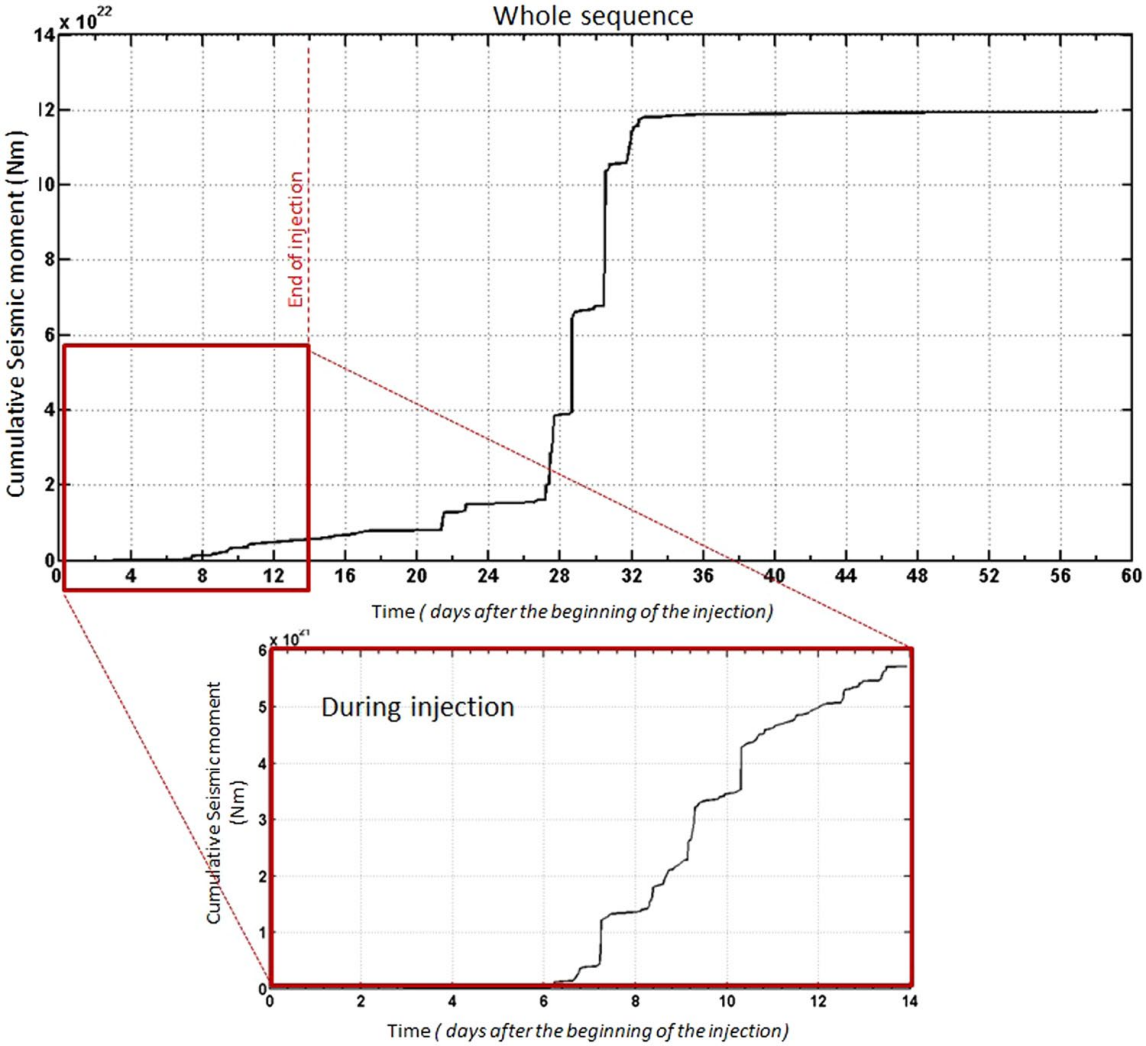
Cumulative seismic moment releases during the whole sequence with a zoom on the cumulative seismic moment releases during the injection. The figure was generated by using the ZMAP software.

We also analyzed the cumulative seismic moment release for the whole sequence taking into account the events with M_w_ ≥ M_c_ (Fig. 9). We observe a clear difference in the slope of the cumulative seismic moment release during and after the stop of injection. The total cumulative seismic moment release is 1.2 × 10^23^ *Nm* (5.8 × 10^21^ *Nm* is released during the injection and the 1.4 × 10^23^ *Nm* remaining is released after the halt of the injection). So, the seismic moment release after the stop of injection operations is about 20 times greater than the one released during the injection. Assuming a constant stress drop scaling, the increase in seismic moment release can be explained as an increased average faults size for events occurring after the stop of injection. This would mean either the activation of pre-existing larger fault segments or the occurrence of multiple ruptures along inter-connected segments due to fluid percolation.

The results illustrated above indicate some variations in the behavior of the sequence during and after the halt of the injection.

### Perspective analysis: Time-dependent parameters estimation

In the retrospective analysis of the seismic catalogue we have identified the b-value of the GR as a useful parameter to discriminate possible differences in the seismicity recorded during and after the stop of the injection. On the other hand, M_c_ depends mostly on the characteristics and noise conditions. However, M_c_ estimation is important to interpret correctly the variations in b-value.

Hereinafter, we explore the potential predictability of these parameters to monitor field operations at a short-term time scale (e.g. days). To this aim, we evaluate M_c_ and b-value as function of time using a sliding window approach. The first critical issue is to identify the optimal time window and the percentage of overlapping between successive windows required to perform the time-dependent estimation of the different parameters.

**Figure 10:**
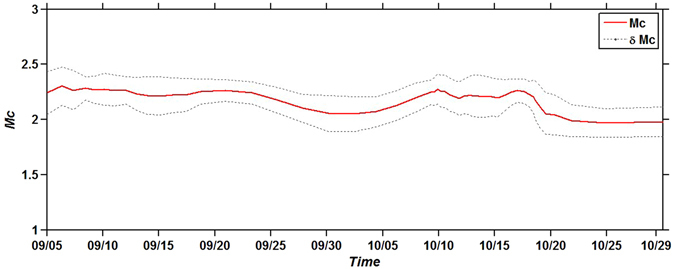
M_c_ as function of time for a sample window of 50 events and 10% of overlapping. The figure was generated by using the ZMAP software.

For the computation of M_c_, based on different trials we have found that, an optimal sample window is 50 events with a 10% of overlapping. Note that, the use of smaller window length with less overlapping could result in the faster prediction. In each window, the maximum curvature method is used to compute M_c_, which is assigned to the middle time of the corresponding window. The final results, which are shown in Fig. 10, indicate that the M_c_ values are comparable during and after injection, without significant changes as function of time. The mean value of the M_c_ for the whole sequence is around 2.2 ± 0.1. This result is coherent with the M_c_ value obtained in the previous retrospective analysis.

For estimating the b-value as function of time, the same sliding window approach is used. We apply the maximum curvature method using only the events with magnitude M_w_ larger or equal to the estimated value of M_c_ = 2.2 (a total of 286 events is considered). We verify with different tests that, in order to obtain a reliable estimation of the b-values as function of time, at least 60 events (size of the sample window) with a 20% of overlapping is needed.

**Figure 11:**
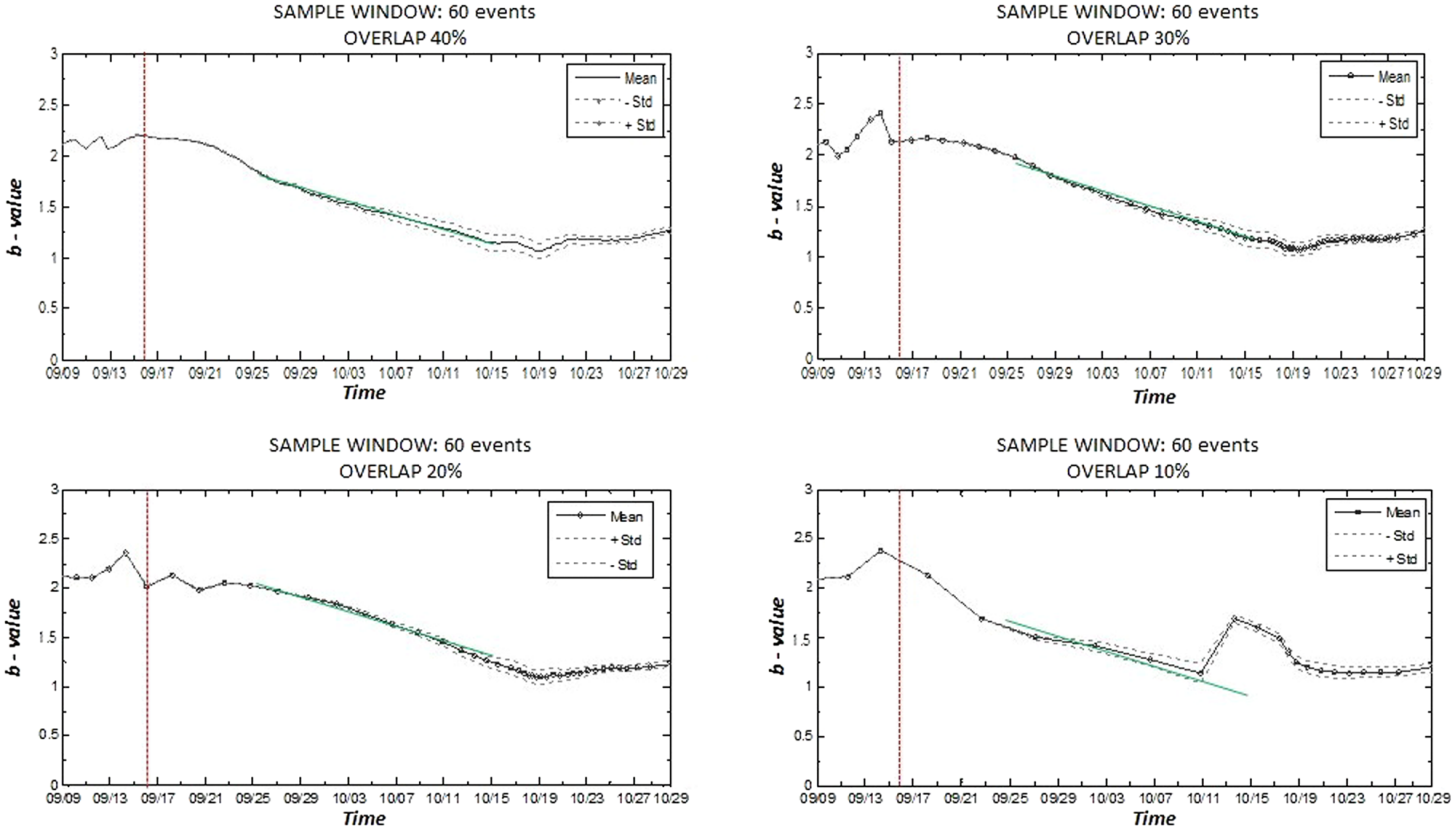
Analysis of the b-value as function of time with a window of 60 events and changing the overlapping between windows. The dotted-line represents the mean b-values and the dashed lines their variability (one standard deviation). The vertical red dash line represents the day when the injection stopped. The straight green line is the slope that characterizes the changes of the b-values. The figure was generated by using the ZMAP software.

The results in Fig. 11 indicate clear and significant changes in the b-value as function of time, where the larger values occur during the injection period. The slope that characterizes the changes of the b-values from 25^th^ September to 10^th^ October (straight green line) remains stable for overlap values of 40%, 30% and 20%, but it changes significantly when percentage of overlap reduced to 10% only. In this last case, some irregularities in the results start to appear and the obtained b-values seem to be less robust. This analysis suggests that for the analyzed catalogue, the minimum number of events to compute stable values of M_c_ and b-value as function of time is 50 events and 60 events respectively. Based on the interpretation of the b-value vs time plot, the observed decrease of the b-value indicates a higher probability of larger magnitude events occurrence with respect to the smaller ones, as those actually observed after the stop of the injection.

While M_c_ value is kept constant along the time, b-value shows a significant time variation, reaching values around 2.0 during the injection period and decreasing to values close to 1.0 after the end of the injection. These results are consistent with those resulting from the previous retrospective analysis.

### Maximum magnitude estimation

In studies dealing with induced seismicity, one of the most debated issues concerns the estimation of the maximum magnitude value (M_max_) associated with events that could be induced by field operation (e.g. refs 25–27). In the present study, we use different statistical techniques to estimate the expected value of M_max_ that can be induced by the anthropogenic activities in Castor.

To this aim, we use a Matlab based computer code provided by Kijko^9^ and Kijko and Singh^28^. Depending on the available information, the estimate of M_max_ can be performed by using a seismic catalogue or using only the information about maximum and minimum magnitude observed in the sequence. Due to the aim of the present study, we have used the first approach, which also requires the b-value, the seismicity rate value and M_c_.

Most of the estimation techniques use parametric estimators but we also consider one non-parametric estimator. The considered procedures are: Tate-Pisarenko (T-P), Kijko-Sellevoll (K-S), Tate-Pisarenko-Bayes (T-P-B), Kijko-Sellevoll-Bayes (K-S-B), non-parametric with Gaussian kernel procedure (N-P-G), procedure based on a few largest earthquakes, Robson-Whitlock (R-W) and Robson-Whitlock-Cooke (R-W-C) (see Kijko and Singh^28^ and reference therein for the details). The main equations of each method are summarized in Appendix A.

The inferred M_max_ values (with associated standard deviations) for each analyzed period are shown in Table 4.

**Table 4:** Summary of M_max_ estimations for each analyzed period with the different approaches applied together with the observed ones in each period (first line in the Table).

	During Injection	After Injection	Whole Sequence
Observed Magnitude	3.2	4.2	4.2
T-P	5.1 ± 1.9	5.5 ± 1.3	6.0 ± 1.8
K-S (Cramer’s approx)	3.5 ± 0.3	4.6 ± 0.4	4.6 ± 0.5
K-S (exact solution)	3.5 ± 0.3	4.6 ± 0.4	4.6 ± 0.5
T-P-B	4.1 ± 0.9	4.7 ± 0.6	4.9 ± 0.7
K-S-B	3.5 ± 0.3	4.5 ± 0.3	4.5 ± 0.3
N-P-G	3.3 ± 0.2	4.3 ± 0.1	4.3 ± 0.7
Based on largest obs	3.3 ± 0.1	4.3 ± 0.1	4.2 ± 0.7
R-W	3.5 ± 0.4	4.3 ± 0.2	4.3 ± 0.2
R-W-C	3.3 ± 0.2	4.2 ± 0.1	4.2 ± 0.2

**Figure 12:**
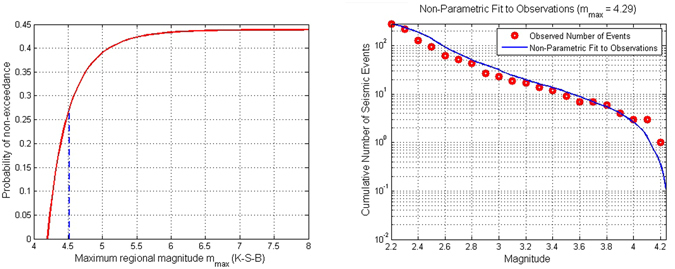
Graphical results of the M_max_ estimation with the K-S-B technique (left panel) and the Non-parametric approach (right panel) taking into account the whole sequence.

We note that all the adopted procedures provide estimates of M_max_ greater or equal than the observed ones. The the T-P procedure significantly overestimate the observed M_max_. The results obtained with the other procedures agree with the observed M_max_ in each analyzed period. The K-W-C procedure provides with the most consistent M_max_ estimations. As example, in Fig. 12 we represent the graphical results of two of the techniques applied, K-S-B as an example of parametric method and the non-parametric approach.

In addition to the previous estimations, we also apply the methodology proposed by Makropoulos and Burton^29^. This methodology assumes that the total accumulated and released energy in a seismogenic region remains fairly constant in a specific time period.

Based on this assumption, a graphical method is proposed, in which the cumulative energy released is plotted as function of time with a straight line that connects the zero energy with the total energy (SS’), being its slope the annual rate of energy released. Since the total energy that may be accumulated and released in a given area is taken as constant, the two lines enveloping maximum and minimum energy released should run parallel to each one and to the SS’ (see ref. 29). Thus, the vertical distance between these two parallel lines enveloping the released energy correlates with the upper limit of the energy that would be observed in the region and it is used to estimate the M_max_, if the accumulated energy during the time was released by a single earthquake (e.g. refs 25, 27). The estimated M_max_ in this case is 4.6 for the whole sequence as well as for the period after the stop of injection and M_max_ = 3.1 during the injection The resulting maximum magnitude values obtained with each methodology considered are summarized in Table 5.

**Table 5:** Observed maximum magnitude and estimated maximum magnitude by Makropo﻿ulos and Burton approach^29^ for each analyzed period.

	M_max_ Observed	M_max_ estimated ﻿(Makropoulos and Burton)
During Injection	3.2	3.1
After Injection	4.2	4.6
Whole Sequence	4.2	4.6

In this case, Makropoulos and Burton^29^ method provides almost similar results for the estimated M_max_ during the injection period. But the magnitude M_max_ 4.6 obtained for the period after the injection, as well as, when the whole sequence is considered, is never actually reached.

## Discussion and Conclusions

In this paper we analyze the observed seismicity using a retrospective and a perspective approach based on the earthquake data catalogue recorded during the gas injection operations at the Castor reservoir by the Spanish National Network.

The main aim was to understand whether the statistical analysis of the recorded seismic sequence during and after gas injection operation could be used to discriminate significant seismicity changes relative to the background and to injection operations, as well as to explore the predictability of some statistical parameters that could be used as a proxy in the future to guide the field operations.

It is worth noting that Castor site is located in a zone of low seismicity and low seismic hazard^30^, (see Fig. 1), but nearby a system of faults with a high seismogenic potential. These are slow faults with speeds ranges from 0.01 mm/y to 0.15 mm/y that may be reactivated due to changes of induced stresses^14–17^. Cesca *et al*.^7^ excluded the possibility that the largest fault in the vicinity of the gas injection wells (Eastern Amposta Fault) were reactivated. Nevertheless, the authors pointed out that a natural cause for the earthquakes cannot be completely excluded, although it is unlikely as from the historical seismicity in the region.

Our statistical analysis provides evidence for two clusters, characterized by a relative change of the b-value and the cumulative seismic moment. In particular, the first cluster occurred during the injection phase and possibly extending after the injection, is characterized by relatively high values of b (around 2.0) and a maximum magnitude of 3.2. The second cluster, after the injection is characterized by a significantly lower b-value and high cumulative seismic moment (a factor 20 higher than for the first cluster). Based on these evidences we argue that while the first cluster can be mainly originated by stress induced by fluid injection, the second cluster is triggered by fault activation due to local stress perturbations as a response to pore-fluid pressure increase.

**Figure 13:**
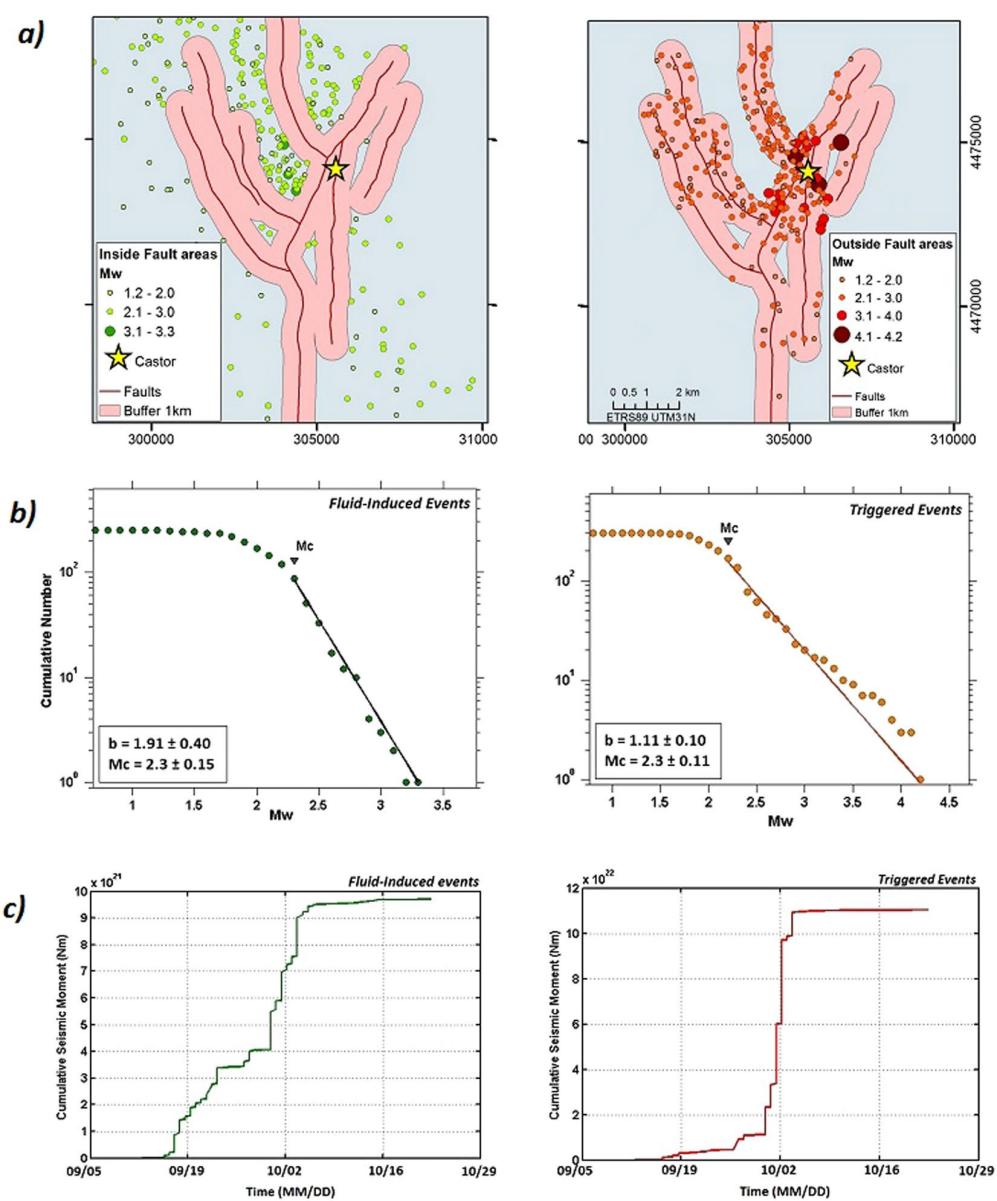
(**a**) Location maps of fluid-induced and triggered events. The criterion used to discriminate between both types of seismicity is their locations nearby/far from mapped faults (events outside a buffer area of 1 km around mapped faults are considered fluid-induced events and the triggered events are those inside the 1 km area around mapped faults). ﻿﻿Fault traces have been digitalized from Cesca *et al.* ^7^﻿. Figure generated with ArcGIS for desktop, version 10.1. (**b**) Gutenberg-Richter laws for both types of seismicity and (**c**) Cumulative seismic moment releases from the fluid-induced and triggered events respectively. These figure were generated by using the ZMAP software.

To support this hypothesis we defined a criterion to discriminate fluid-induced and triggered events based on their location nearby/far away from known mapped faults embedded in the volume surrounding the injection site and gas storage (Fig. 13a).

Next, we estimated the completeness magnitude Mc, the b-values and cumulative seismic moment release as inferred from the two catalogs which included the two event types (Fig. 13b and c).

This analysis confirms that near-fault triggered events are characterized by a lower b-value and a faster seismic moment release than fluid-induced earthquakes. The acceleration of the cumulative seismic moment for triggered events could be explained by a general increase of the fault size (in the hypothesis of a constant stress drop scaling with magnitude).

The results obtained by the time dependent analysis, show that the M_c_ as function of time is quite constant (it depends on the network and noise conditions), whereas the b-value is changing clearly with the time featuring higher values during the injection period and lower values after the stop of injection with a consequent increase in the probability of occurrence of larger magnitude events. Hence, given an appropriate time window, the b-value could be used as a proxy for monitoring the possible evolution of the induced toward triggered seismicity. For the analyzed sequence we find that the minimum number of events to be used for estimating a reliable b-value as function of time is 60 events with an overlapping of 20% between sample windows. However, a denser seismic network, with a larger accuracy in the locations of events, would allow to sensibly reducing this sample window, with a resulting faster prediction.

As for the expected maximum magnitude, several approaches are applied in each considered time period (i.e., during and after the injection and the whole sequence) by using the methods of Kijko^9, 28^. In addition, the Makropoulos and Burton^29^ method is also applied. In general, most of the procedures for the assessment of M_max_ provide values consistent with the observed M_max_, except for the T-P procedure, which provide the greatest estimation with a large standard deviation. The procedure that better estimates the actually observed M_max_ values is the Robson-Whitlock-Cooke (R-W-C) procedure.

Finally, we show that the time-varying statistical analysis of the earthquake catalogue recorded during industrial operations in a fluid injection extraction area may help to rapidly identify ongoing anomalies in the rate of occurrence and magnitude distribution of earthquakes and possibly to detect the re-activation of nearby faults, producing higher magnitude seismicity. This work has been solely based on the earthquake catalogue from the national, large scale seismic network operated by IGN. To get a complete picture on the ongoing fracture phenomena, a more refined real-time analysis of seismicity should include the accurate estimation of source parameters (e.g. the earthquake location, fault mechanism, seismic moment and stress release) and the timely analysis of their spatial and time distribution. The next step could therefore be to combine all this information in a decisional scheme which provides the basis for a traffic-light system to be used for controlling and regulating the field operations as a function of the observed changes in seismicity occurrence. One possibility is to assess a threshold-based decisional scheme, which uses the contemporary observation of a significant decrease of b-value and increase of the cumulative moment release, issuing an alarm when pre-defined rate levels are exceeded. The latter must be based on a calibration of the system by analyzing in detail the background seismicity and its statistical properties.

## Electronic supplementary material

Supplementary Information
