# Supplementary material for: Temporal evolution of a seismic sequence induced by a gas injection in the Eastern coast of Spain
Source: Sci Rep. 2017 Jun 6;7:2901. doi: 10.1038/s41598-017-02773-2 (PMC5460170; doi:10.1038/s41598-017-02773-2)

Temporal evolution of a seismic sequence induced by a gas injection in the Eastern coast of Spain

Sandra Ruiz Barajas:
[1.] Geodesia y Cartografía, Campus Sur UPM, Universidad Politécnica de Madrid, ETSI Topografía, Ctra. de Valencia, km 7.5, 28031 Madrid, Spain.
E-mail: Sandra.ruizb @upm.es

Nitin Sharma:
[2.] Dipartimento di Fisica, Università degli Studi Federico II, Complesso Universitario di Monte S. Angelo, Edificio 6, via Cintia, 80126, Napoli, Italy

[3.] Istituto Nazionale di Geofisica e Vulcanologia Osservatorio Vesuviano, Via iocleziano 328, 80124, Napoli, Italy

*Now at:
[4.] Shillong Geophysical Research Centre, Indian Institute of Geomagnetism, Shillong-793005

Vincenzo Convertito
[3.] Istituto Nazionale di Geofisica e Vulcanologia Osservatorio Vesuviano, Via Diocleziano 328, 80124, Napoli, Italy

Aldo Zollo:
[2]. Dipartimento di Fisica, Università degli Studi Federico II, Complesso Universitario di Monte S. Angelo, Edificio 6, via Cintia, 80126, Napoli, Italy

Belén Benito Oterino:
[1.] Geodesia y Cartografía, Campus Sur UPM, Universidad Politécnica de Madrid, ETSI Topografía, Ctra. de Valencia, km 7.5, 28031 Madrid, Spain.

Appendix A

Here we summarize the main equations used to estimate the maximum magnitude. A complete derivation of the equations can be found in Kijko and Singh (2011).

Tate-Pisarenko (T-P)

If *n* is the number of the main earthquakes with magnitude larger that m_{min} that occurred in a given time period, assuming a Gutenberg-Richter (GR) frequency-magnitude relationship with a given *b*-value, the T-P technique estimates m_{max} based on the following equation:

$$m_{max} = m_{max}^{obs} + \frac{1-exp[-\beta(m_{max}^{obs}-m_{min})]}{nexp[-\beta(m_{max}-m_{min})]} \text{(A1)}$$

where m_{max}^{obs} is the maximum observed magnitude and $\beta$is *b* ln10. The uncertainty is given by:

$$VAR(m_{max}) = \sigma_M^2 + \frac{n+1}{n^3}\left[\frac{1-exp[-\beta(m_{max}^{obs}-m_{min})]}{nexp[-\beta(m_{max}-m_{min})]}\right]^2 \text{(A2)}$$

where $\sigma_M$denotes the standard error onm_{max}^{obs}.

Kijko-Sellevoll (K-S) (Cramer's Approximation)

In the hypothesis of a GR relationship, this procedure estimates m_{max} using the following equation:

$$m_{max} = m_{max}^{obs} + \frac{E_1(n_2)-E_1(n_1)}{\beta exp(-n_2)} + m_{min}exp(-n) \text{(A3)}$$

where *n* is the number of the main earthquakes with magnitude larger that m_{min} , $E_1(\bullet)$ denotes an exponential integral function defined as $E_1(z) = \int_0^{\infty} exp\,(-\zeta)/\zeta\; d\zeta$and n1, and n2 are given by:

$$n_1 = \frac{n}{1-exp[-\beta(m_{max}-m_{min})]} \text{(A4)}$$

$$n_2 = n_1 exp[-\beta(m_{max} - m_{min})] \text{(A5)}$$

Kijko-Sellevoll (K-S) (Exact solution)

This procedure prescribes that m_{max} is obtained as a solution of the equation:

$$m_{max} = m_{max}^{obs} + \frac{m_{max}-m_{min}+\frac{1}{\beta}\sum_{i=1}^{n}(-1)^i/i\binom{n}{i}[1-exp(-i\beta(m_{max}-m_{min}))]}{[1-exp(-\beta(m_{max}-m_{min}))]^n} \text{(A6)}$$

$$VAR(m_{max}) = \sigma_M^2 + \left[\frac{m_{max}-m_{min}+\frac{1}{\beta}\sum_{i=1}^{n}(-1)^i/i\binom{n}{i}[1-exp(-i\beta(m_{max}-m_{min}))]}{[1-exp(-\beta(m_{max}-m_{min}))]^n}\right]^2 \text{(A7)}$$

where $\sigma_M$denotes the standard error onm_{max}^{obs}.

Tate-Pisarenko-Bayes (T-P-B)

The equation for estimating m_{max} through the T-P-B procedure is as follows:

$$m_{max} = m_{max}^{obs} + \frac{1}{n\beta C_\beta}\left(\frac{p}{p+m_{max}^{obs}-m_{min}}\right)^{-(q+1)} \text{(A8)}$$

being $p = \bar{\beta}/\sigma_{\beta}^{2}$, $q = \left(\bar{\beta}/\sigma_{\beta}\right)^{2}$. The symbol $\bar{\beta}$denotes the known, mean vaue of □□andσ_{β} is the known standard deviation. The coefficient$C_{\beta}$is equal to:

$$C_{\beta} = \{1 - [p/(p + m_{max} - m_{min})]^{q}\}^{-1} \text{(A9)}$$

The corresponding error is given by:

$$VAR(m_{max}) = \sigma_{M}^{2} + \left[\frac{n+1}{n^{3}}\right] \frac{1}{(n\beta C_{\beta})^{2}} \left(\frac{p}{p + m_{max}^{obs} - m_{min}}\right)^{-2(q+1)} \text{(A10)}$$

where σ_{M}denotes the standard error onm_{max}^{obs}.

Kijko-Sellevoll-Bayes (K-S-B)
The Bayesian version of the Kijko-Sellevoll (K-S) described above for estiamting m_{max} is as follows:

$$m_{max} = m_{max}^{obs} + \frac{\delta^{(1/q)} exp[nr^{q}/(1-r^{q})]}{\beta} [\Gamma(-1/q\,, \delta r^{q}) - \Gamma(-1/q\,, \delta)] \text{(A11)}$$

where m_{max}^{obs}, $r = p/(p + m_{max} - m_{min})$, $\delta = nC_{\beta}$, and $\Gamma(:,:)$is the incomplete Gamma Function. The xpression for p and q and $C_{\beta}$are the same as in the (T-P-B) procedure.
The associated uncertainity is given by:

$$VAR(m_{max}) = \sigma_{M}^{2} + \left[\frac{\delta^{(1/q)} exp[nr^{q}/(1-r^{q})]}{\beta} [\Gamma(-1/q\,, \delta r^{q}) - \Gamma(-1/q\,, \delta)]\right]^{2} \text{(A12)}$$

where σ_{M}denotes the standard error onm_{max}^{obs}.

Non-parametric with Gaussian kernel procedure (N-P-G)
This procedure is used when the empirical log-frequency-magnitude relationship does not exhibits linearity. If $\Phi(\xi)$denotes the standard Gaussian cumulative distribution and h is a positive smoothing factor, m_{max} is estimated through:

$$m_{max} = m_{max}^{obs} + \int_{m_{min}}^{m_{max}} \left[\frac{\sum_{i=1}^{n}\left[\Phi\left(\frac{m-m_{i}}{h}\right) - \Phi\left(\frac{m_{min}-m_{i}}{h}\right)\right]}{\sum_{i=1}^{n}\left[\Phi\left(\frac{m_{max}-m_{i}}{h}\right) - \Phi\left(\frac{m_{min}-m_{i}}{h}\right)\right]}\right]^{n} dm \text{(A13)}$$

if the integral on the right member of equation A13 is indicated with □, then the expression of the uncertainty is given by:

$$VAR(m_{max}) = \sigma_{M}^{2} + \Delta^{2} \text{(A14)}$$

where σ_{M}denotes the standard error onm_{max}^{obs}.

Robson-Whitlock (R-W)
Assuming that the data are ordered in ascending order $m_1 \leq m_2 \leq \ldots \leq m_{n-1} \leq m_{max}^{obs}$, m_{max} is estimated by:

$$m_{max} = m_{max}^{obs} + (m_{max}^{obs} - m_{n-1}) \text{(A15)}$$

and its variance is:

$VAR(m_{max}) = 5\sigma_M^2 + (m_{max}^{obs} - m_{n-1})^2$(A16)

Robson-Whitlock-Cooke (R-W-C)

Assuming that the observed magnitudes are sampled from a truncated distribution as the double truncated G-R relationship, and the magnitude values are taken in ascending order ($m_1 \leq m_2 \leq ... \leq m_{n-1} \leq m_{max}^{obs}$), m_{max} is estimated by:

$m_{max} = m_{max}^{obs} + 0.5(m_{max}^{obs} - m_{n-1})$(A17)

and its variance is:

$VAR(m_{max}) = 0.5[3\sigma_M^2 + 0.5(m_{max}^{obs} - m_{n-1})^2]$(A18)

Procedure based on L1-norm fit of CDF

Assuming a GR frequency-magnitude relationship and that m_{min} is known, the technique is based on the minimization of given functional $\Phi(\theta)$where the vector model parameter is given by $\theta = (\beta, m_{max})$. For a set of n odered (in increasing oreder) magnitude value m_i , the functional is ginve by:

$\Phi(\theta) = \sum_{i=1}^{n} \left| F_M(m_i) - \hat{F}_M(m_i) \right|$(A19)

where $|\bullet|$indicates the absolute value and $\hat{F}_M(m_i)$is the empirical distribution function equal to $i/(n+1)$.

Appendix B

Here we summarize the methods used for estimating the magnitude of completeness (Mc) with a special focus on how the uncertainty for all the cases

ENTIRE-MAGNITUDE-RANGE (EMR)

This method (based on Ogata and Katsura, 1993) uses the entire data set, including the range of not completness magnitudes. This allows to maximize the amount of data available for the Mc estimation in order to achieve a most stable and robust result, creating a comprehensive seismicity model. The method consists of two parts: one to model the complete part, and one to sample the incomplete part of the frequency-magnitude distribution. For data above an assumed Mc, it is presume a Gutenberg-Richter power-law behaviour. The a and b values of the Gutenberg and Richter law are computed using a maximum-likelihood estimate. For data below, a normal cumulative distribution fuction is assumed q(M| μ, σ). This function describes the detection capability as a function of magnitude is fitted to the data. The probability of a seisic network to detect an earthquake of a certain magnitude is reflected by q(M| μ, σ) and can be expressed as.

$$q(M|\, \mu, \sigma) = \begin{cases} \frac{1}{\sigma\sqrt{2\pi}} \int_{-\infty}^{Mc} \exp\left(-\frac{M-\mu^2}{2\sigma^2}\right) dM, M < Mc \\ 1 \qquad , M \geq Mc \end{cases}$$

Here, μ is the magnitude at which 50% of the earthquakes are detected and σ represents the standard deviation describing the width of the range where earthquakes are partially detected. Higher values of σ indicate that the detection capability of a specific network decreases faster. Earthquakes with magnitudes equal to or greater than Mc are assumed to be detected with a probability of one. The free parameters μ and σ are estimated using a maximum-likelihood estimate (Woessner and Wiemer, 2005).

The EMR method demands more computation time than other methods and it should be appliend only when the incomplete part of the catalogue is available.

MAXIMUM CURVATURE-METHOD (MAXC)

Method proposed by Wiemer and Wyss (2000) and based on the assumption of self-similarity. This method estimate Mc in a fast and reliable way, by defining the point of the maximum curvature (MAXC) as magnitude of completeness and computing the maximum value of the first derivative of the frecuency-magnitude curve.
In practice, this matches the magnitude bin with the highest frequency of events in the non-cumulative frequency-magnitude distribution.

Although, this method is easy to apply and provides a relative robust estimations applicability and relative robustness of this method, Mc is often underestimated especially for gradually-curved frequency–magnitude distributions that result from spatial or temporal heterogeneities.

GOODNESS-OF-FIT (GFT)

In order to estimate Mc, the goodness-of-fit method compares the observed frequency-magnitude distribution with synthetic ones (Wiemer and Wyss, 2000). The GFT is computed as the absolute difference of the number of events in the magnitude bins between the observed and synthetic Gutenberg-Richter distribution.
Synthetic distributions are calculated using estimated a and b values of the observed dataset for M ≥ Mco as a funcion of ascending cutoff magnitudes Mco.
The fit in percentage to the observed frequency-magnitude distribution is defined by R, and is computed as a function of cutoff magnitude. A model is found at and R-value at which a predefined percentage (90%-95%) of the observed data is modeled by a straight line. Note that, for real catalogues, the 95% level of fit is rarely obtained and the 90% level is a compromise

For estimating the uncertainty of Mc, Monte Carlo approximation of the bootstraps method is used (Efron 1979, Chernick, 1999), which can be combined with all methods used and described above.
Bootstraps sample earthquake catalogues are generated y drawing with replacement an equivalent amount of events from the original catalogue. For each of the bootstraps sample earthquake catalogues, Mc and b are calculated. The second moment of the evolvingempirical distribution of Mc and b values is defined as the uncertainty of Mc and b, respectively.

Reference

Chernick, M.R. (1999). Bootstrap methods: A practioner's guide, in Wiley Series in Probability and Statistics, W.A. Shewhart (Editor), Wiley and Sons, Inc., New York

Efron, B. 1977 Rietz Lecture, Bootstrap Methods-Another Look at the Jackknife, Ann. Statist. 7, 1-26 (1979).

Kijko A., and M. Singh (2011). Statistical Tools for Maximum Possible earthquake magnitude estimation, Acta Geophysica, vol. 59, no.4, pp. 674-700, DOI:10.2478/s11600-011-0012-6.

Ogata, Y. and Katsura, K. Analysis of temporal and spatial heterogeneity of magnitude frequency distributio inferred from earthquake catalogs, Geophys. J. Int. 113, 727-738 (1993)

Wiemer, S. & Wyss.M. Minimum magnitude of complete reporting in earthquake catalogs: examples from Alaska, the western united states, and japan. Bull. Seismol. Soc. Am. 90, 859–869 (2000).

Woessner, J. & Wiemer, S. Assessing the quality of earthquake catalogues: Estimating the magnitude of completeness and its uncertainty. *Bull. Seismol. Soc. Am.* **95, no. 2,** 684–698 (2005).

Figures

Figure S1.Results of GR laws including whole sequence, during injection, and after injection for each of the method used to estimate Mc value (from top to bottom: EMR, Maximum Curvature and Goodness of fit)

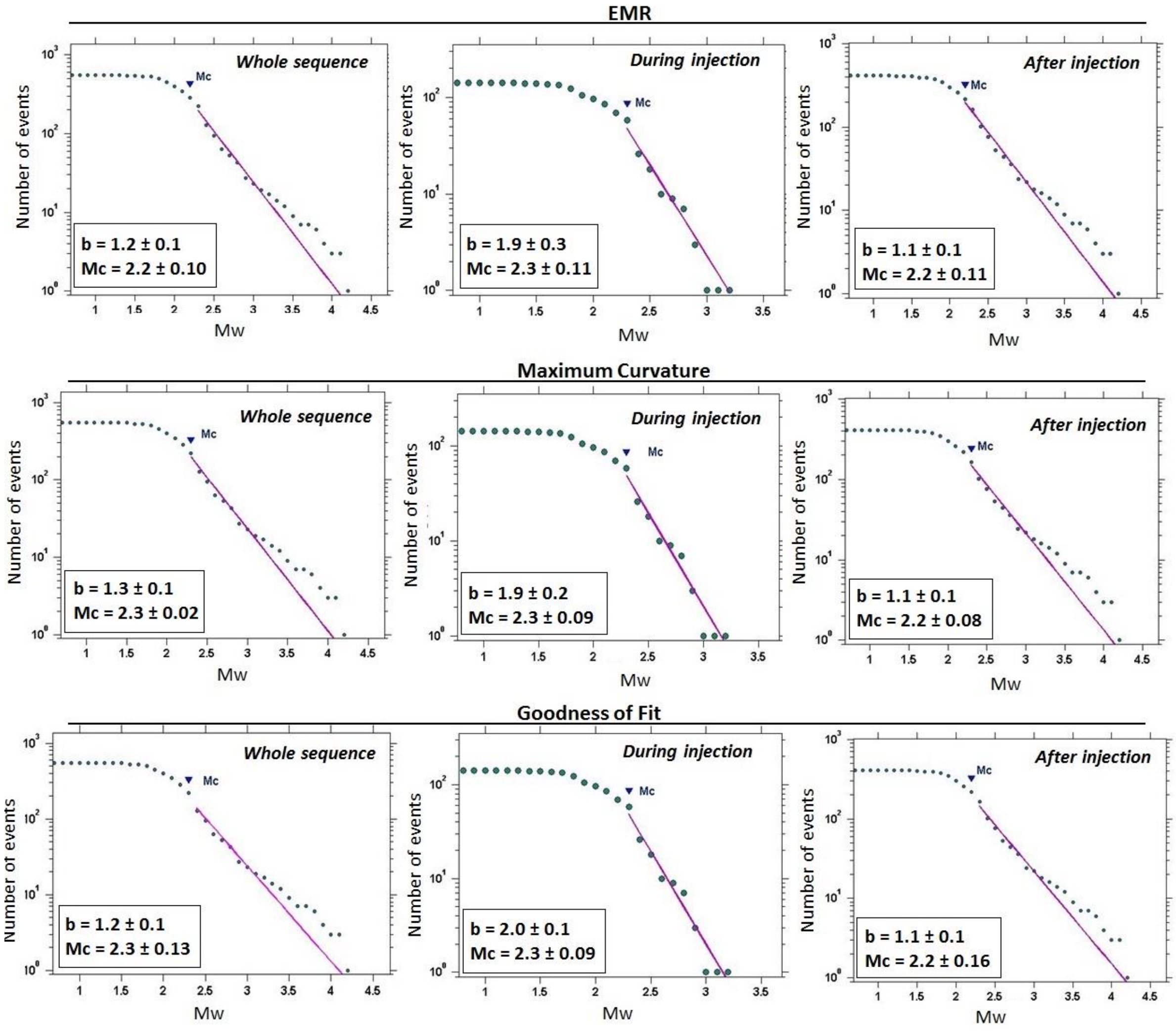

Supplement: Supplementary file 1 — Supplementary Information [file 41598_2017_2773_MOESM1_ESM.pdf]
